# Amyand’s Hernia: A Rare Surgical Pathology of the Appendix

**DOI:** 10.7759/cureus.2827

**Published:** 2018-06-18

**Authors:** Shimron I Bhatti, Muhammad Usman Hashmi, Usman Tariq, Haran Innocent Bhatti, Julius Parkash, Zainab Fatima

**Affiliations:** 1 Orthopaedics, Shifa International Hospital, Islamabad, PAK; 2 Thoracic Surgery, Nishtar Medical University Hospital, Multan, PAK; 3 Research Assistant, Yale University School of Medicine, New Haven, USA; 4 Surgery, Shifa College Of Medicine, Islamabad, PAK; 5 Surgery, Christian Hospital Taxila, Taxila, PAK; 6 Medicine, Shifa International Hospital, Islamabad, PAK

**Keywords:** amyand's hernia, appendix, hernia, appendectomy, amyand's hernia, inguinal, darn repair

## Abstract

Amyand’s hernia is characterized by the presence of an inflamed, non-inflamed or perforated appendix within the sac of an inguinal hernia. This is an exceedingly rare presentation and most of the cases are diagnosed incidentally during surgery. Here we describe a case of an Amyand’s hernia in a patient who presented in our outpatient clinic with an irreducible right-sided inguinal hernia. There were no signs of ischemic complications. During the surgery, an appendix was found within the hernial sac. An uneventful appendectomy along with a tension-free darn repair was performed. The current case report emphasizes that this pathology must be kept in mind while treating a patient with a right-sided inguinal hernia.

## Introduction

An Amyand’s hernia is a clinical phenomenon characterized by the appendix ensnared within an inguinal hernia. This is a rare clinical occurrence that is mostly observed during a surgical procedure [1]. We report a case of this rare clinical entity, following the patient’s presentation in our clinical setting.

## Case presentation

A 24-year-old female presented to our surgical outpatient department with the complaint of an asymptomatic swelling in the right inguinal region for the past two years. Her clinical predicament subsequently worsened with the onset of a sharp stabbing pain in her right inguinal region, with associated nausea and vomiting for two days. She also provided a history of a low-grade fever. There was no history of abdominal distention and constipation. Initial assessment found the patient to be alert, well-oriented and in no apparent distress. Her heart rate was 98 beats per minute with a blood pressure of 130/75 mm Hg, a respiratory rate of 16 per minute and a temperature of 99.4°F. An abdominal exam revealed no scar-mark of a previous cesarean section. She was found to have a non-distended abdomen with tenderness in the right inguinal region. A 5 cm x 7 cm irreducible mass with normal overlying skin was noted in the right inguinal region with an expansile cough reflex. All other hernial orifices were intact. Normal bowel sounds were perceptible on auscultation. A digital rectal exam revealed an empty rectal vault. Per our clinical exam, the patient was diagnosed as a usual case of a right-sided inguinal hernia. She was admitted and scheduled for an open herniorrhaphy. The details of her pre-operative investigations are described in Table 1.

**Table 1:** Pre-operative laboratory investigations. CRP: C-reactive protein; aPTT: Activated partial thromboplastin time; PT: Prothrombin time; ELISA: Enzyme-Linked Immunosorbent Assay; HBsAg: Hepatitis B surface antigen.

Initial Investigation	Value	Reference Range
Hemoglobin	9.8 g/dL	Female: 12-16.0 g/dL
Hematocrit	28.7%	Female: 36-46%
Reticulocyte count	1%	Adults and Children, 0.5-2.5%
Platelet count	160,000/µL	150,000-400,000/µL
Total leukocyte count (TLC)	14,500 cells/µL	4500-11,000/µL
CRP	49 mg/dL	Up to 5.0 mg/L
Serum Sodium	139 mEq/L	Adults 136-145 mEq/L
Serum Potassium	4.2 mEq/L	3.5-5.1 mEq/L
aPTT	33 seconds	25-40 seconds
PT	12 seconds	11-15 seconds
Anti-Hepatitis C antibodies by ELISA	Negative	
HBsAg by ELISA	Negative	

**Figure 1:**
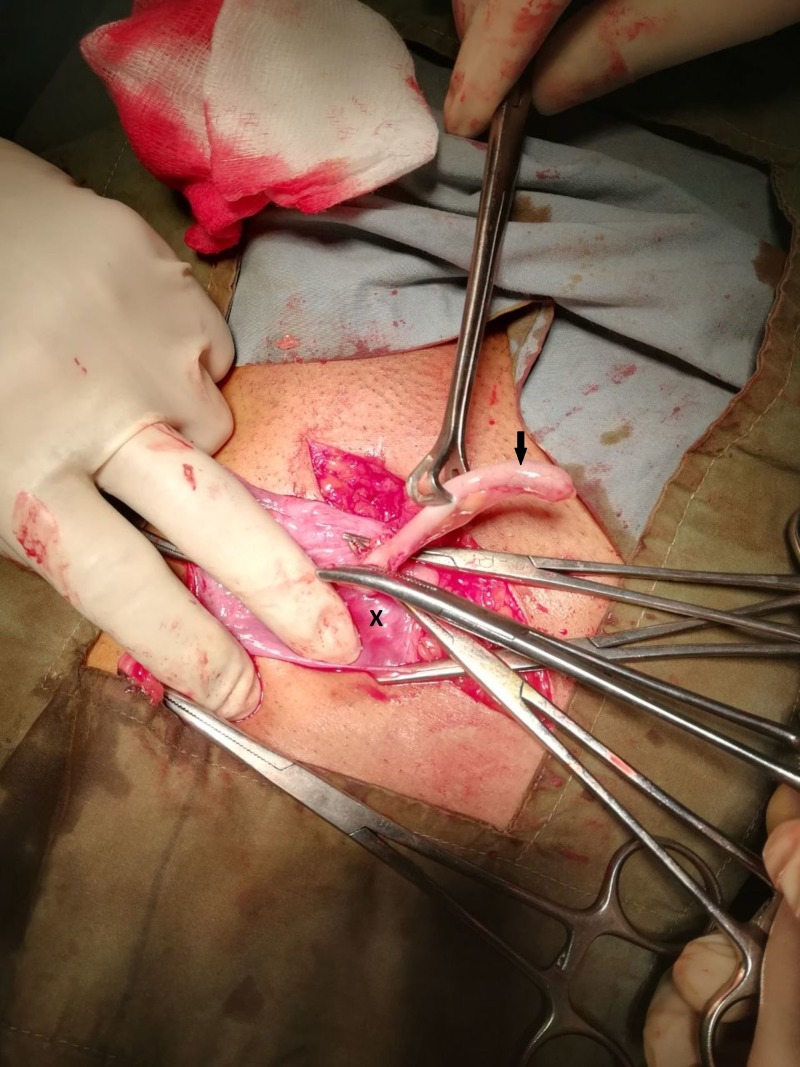
An edematous appendix (black arrow) emerging from within the sac (marked by X) of an inguinal hernia.

Intraoperatively, a 10-cm incision was made in the right inguinal region and the hernial sac was approached. Upon opening the sac, the appendix was discovered within the hernial pouch. On gross examination, the appendix was enlarged, edematous and approximately 10 cm in length. Figure 1 shows our intra-operative findings.

Consequently, an appendectomy was performed followed by a tension-free darn repair. The postoperative period was uneventful and our patient has had a favorable clinical outcome to date.

## Discussion

An Amyand’s hernia is a rare clinical entity. It is defined as an inguinal hernia which ensnares an inflamed, non-inflamed or perforated appendix [2]. Medical literature states that this uncommon presentation occurs in only 1% of all inguinal hernias which signifies the rarity of this clinical presentation [1]. Furthermore, an inflamed appendix is found in only 0.07–0.13% cases of an inguinal hernia [3]. One of the earliest documented cases of an appendectomy on a human subject was secondary to an Amyand’s hernia. The surgery was performed by an English surgeon, Claude Amyand, in 1735. This historical case involved a fistulous communication between the hernial sac and the overlying skin between the thigh and the scrotum [4].

Losanoff and Basson provided the original classification of the different types of Amyand’s hernia and detailed the proposed means for managing patients presenting with the varying subtypes. Table 2 describes the detailed information about this classification system [2, 5, 6].

**Table 2:** A classification of the different types of Amyand’s hernia with a recommended plan of management for each subtype.

Classification	Description	Management
Type I	Normal appendix enclosed within the sac of an inguinal hernia	Reduce the hernia, with a choice of performing an appendectomy in young patients, followed with a mesh hernioplasty
Type II	Patient has signs and symptoms of acute appendicitis, but there is no evidence of abdominal sepsis	Perform an appendectomy followed by a hernioplasty
Type III	Patient has signs and symptoms of acute appendicitis, with evidence of abdominal sepsis	Perform an appendectomy followed by a hernioplasty
Type IV	Patient has signs and symptoms of acute appendicitis in the setting of an affiliated abdominal pathology outside the hernial sac	Perform an appendectomy followed by a hernioplasty. A subsequent follow-up for the affiliated pathology should be undertaken after the procedure

Singal and Gupta put forth a revised adaptation of this classification by considering the presence of a previous abdominal incision, which could lead to a resultant incisional hernia. This led to the proposal of another subtype, Type V, which was further subdivided into three categories. Subtype VA was described as a normal appendix within an incisional hernia which should be managed as Type I. Subtype VB was described as a case of appendicitis ensnared within the sac of an incisional hernia and should be managed via an appendectomy through a hernia followed by a mesh hernioplasty repair. And Type VC was described as a case of appendicitis within the incisional hernial sac with a concomitant abdominal pathology which should be managed analogously to Type IV [2, 7]. Per these classifications, our patient had a Type II Amyand’s hernia. Consequently, an appendectomy was performed followed by a tension-free darn repair.

It should be noted that most cases of Amyand’s hernia are diagnosed intraoperatively. While pre-operative imaging modalities such as an ultrasound or computed tomography (CT) scans may pave the way for a proper diagnosis, performing these tests is not a part of the routine practice in the setting of an uncomplicated inguinal hernia, which is usually a clinical diagnosis [8]. Consequently, most cases of Amyand’s hernia are undiagnosed pre-operatively and treated as mere inguinal or inguinoscrotal swellings.

## Conclusions

Amyand’s hernia is a rare clinical occurrence and diagnosed intra-operatively in most clinical cases. A pre-operative diagnosis via an ultrasound or CT imaging is possible but has minimal functionality in the setting of an uncomplicated inguinal hernia which is a routine clinical diagnosis. Most patients are managed with an appendectomy followed by a hernial repair with a resultant resolution of symptoms.
